# Virtual reality for pain and anxiety of pediatric oncology patients: A systematic review and meta-analysis

**DOI:** 10.1016/j.apjon.2022.100152

**Published:** 2022-09-28

**Authors:** Zhi Cheng, Shanzhen Yu, Wen Zhang, Xinxin Liu, Yijin Shen, Hong Weng

**Affiliations:** Zhongnan Hospital of Wuhan University, Wuhan, China

**Keywords:** Virtual reality, Pediatric oncology patients, Pain and anxiety, Systematic review

## Abstract

**Objective:**

The aim of this paper is to systematically evaluate the effects of virtual reality (VR) on pain, anxiety, and fear symptoms of pediatric patients with cancer.

**Methods:**

PubMed, Web of Science, Embase, Cochrane Library, Scopus, CINAHL, and four Chinese medical databases were searched from January 1, 1975, to February 22, 2022. Randomized controlled trials on the effects of VR technology on pediatric cancer patients were searched. Two researchers independently screened literatures, extracted data, and evaluated literature quality according to inclusion and exclusion criteria, and meta-analysis was performed by RevMan 5.3.

**Results:**

A total of 379 children with cancer were included in six randomized controlled trials. Overall, there were significant differences in favor of VR in pain (MD ​= ​−4.82, 95% CI [-7.74, −1.89], *P*＜0.01; *I*^*2*^​= ​95%, *P*＜0.01), anxiety (SMD ​= ​−1.47, 95% CI ​[​−2.46, −0.48], *P*＜0.01; *I*^*2*^ ​= ​92%, *P*＜0.01), and fear (MD ​= ​−1.25, 95% CI [-1.78,0.72], *P*＜0.01; *I*^*2*^ ​= ​0%, *P* ​= ​0.69).

**Conclusions:**

VR is beneficial to improve the pain, anxiety, and fear mood of pediatric cancer patients. Larger sample sizes and more rigorous studies are needed.

## Introduction

Cancer is a common and debilitating disease and a leading cause of death among children.^1^ The estimated global incidence of childhood cancer in 2015 was 396,670 cases.^2^ In recent years, due to the improvement of diagnostic methods, the rate of early diagnosis of childhood tumors has significantly increased. At present, the 5-year survival rate of children under 15 years old in the United States has reached 70% or higher.^3^ Children diagnosed with cancer experience many painful and often anxiety-inducing medical procedures, such as venipuncture, bone marrow, and lumbar puncture.^4^ Besides, anxiety and fear are common negative emotions in children with cancer except pain. Many children are full of fear of various medical operations, full of anxiety about the unknown development of the disease, and afraid that they are different from other children.^5^ And pain will add anxiety and fear to the child. There is a certain relationship between pain, anxiety, and fear in patients, but it is hard to tell which is the cause and which is the effect.^6^

Significant progress has been made in the prognosis of children with cancer.^7^ Nevertheless, pediatric operative pain is still one of the most difficult symptoms to manage in hospitals. When feeling pain, most children with cancer often show crying, twisting, and muscle stiffness.^8^ Studies have shown that 50%–60% of the children with cancer reported obvious pain and anxiety when receiving various medical operations, and nearly 63.0% of the children with cancer further developed fear of doctors and nurses.^9^ Common nonpharmaceutical methods helping children relieve pain include massage, touching the pain area, playing interesting games and movies, and telling children stories about overcoming difficulties to build confidence.^10^ Medications used to relieve medical pain in children include topical skin creams, acetaminophen, tylenol, bisacetaminophen, and bunoprofen.^11^^,^^12^ Moderate to severe pain can be obtained with prescription painkillers such as morphine, oxycodone, codeine, and so on.^13^

When children with cancer feel pain, anxiety, or fear, medical staff have to spend extra time to calm them, and parents may even coerce and restrain them to complete medical procedures.^14^ These behaviors not only cause discomfort for oncology pediatrics, increase the time required for operation, and reduce the success rate of medical operation but also may cause tension in doctor––patient relationship and treatment resistance.^15^ During the treatment of cancer, children frequently have fear of unfamiliar environment and separation anxiety, which increased children's perception of pain, anxiety, and fear.^16^^,^^17^ Consequently, there is an urgent need to explore more effective and nonpharmacological therapies to decrease pain, anxiety, and fear during medical procedures.

Virtual reality (VR) technology is a new intervention method applied in medical field, which based on the principle of distraction, providing real perceptual stimuli such as visual images, spatial sounds, tactile, and olfaction feedback stimuli.^18^ Virtual reality technology makes full use of the individual's limited attention cognitive resources, moving the individual's attention from the “real world” to the “virtual world” creating a sense of complete immersion^19^, ^20^, ^21^. VR will stimulate as many senses as possible to reduce the patient's perception of pain and anxiety by disrupting awareness and sensitivity to stressors, reallocating attention, concentration, emotional input, redirecting neural signals, and shifting harmful stimuli to other neutral or pleasant events.^22^ VR provides a noninvasive method for pain and anxiety management through the principle of distraction.^23^

At present, VR technology has been widely used in perioperative anesthesia induction, burns, stomatology, autism, brain injury rehabilitation, and other fields, which have shown that VR technology can improve pain and anxiety in children^24^, ^25^, ^26^, ^27^, ^28^, ^29^. Previous systematic reviews focused more on the impact of VR technology on children with certain situation like tooth extraction or injection or analyzed the symptoms of pain and anxiety in children, while the meta-analysis of VR technology on children with cancer has not been studied yet.^6^^,^^15^ Therefore, evaluating the effect of VR technology on relieving pain and anxiety related to children with cancer is needed in order to provide scientific evidence-based basis for relieving pain and anxiety in clinical nursing practice.

## Methods

### Study design

In this study, the PRISMA standard of the Cochrane Handbook of Interventional Reviews was used for study design, screening, and reporting. The protocol was registered in the INPLASY database with the registration number: 202230108.

### Search strategy

Two reviewers independently searched the following databases: Pubmed, Embase, Cochrane Library, Web of science, Scopus, Cinhal, the Chinese Biomedical Database (CBM), the China National Knowledge Infrastructure (CNKI), VIP Journal Integration Platform, and Wanfang Med Online, without language restriction, from January 1, 1975, to February 22, 2022. A combination of subject words and free words was adopted. The retrieval strategy was determined after repeated retrieval, supplemented by manual retrieval, and the published systematic evaluation about virtual of cancer were traced. The following Medical Subject Heading terms and text words were used: “virtual reality,” “digital technology,” “VR,” “virtual reality exposure therapy,” “smart glasses,” “children,” “pediatric,” “kid,” “toddler,” “boy,” “girl,” “infant,””cancer,” “neoplasms,” “oncology,” “tumour,” and “malignancy.”。

### Inclusion and exclusion criteria for study selection

**Inclusion criteria: Types of studies:** Randomized controlled trials were included with restriction of language only for Chinese and English. **Types of participants** aged 18 years or younger clinically diagnosed as any types of cancer identified by pathological or cytological diagnosis. **Types of intervention** groups received VR in regardless of combining other treatments or not, without restricting on VR's duration, frequency, and modalities. The control group undergoes standard care, no intervention or some treatment differed from VR. **Types of outcome measures:** The outcomes were pain, anxiety, and fear.

**Exclusion criteria:** Articles other than English or Chinese; articles with no access to the full text; articles published more than 5 years ago.

### Quality appraisal

The methodological quality based on methods endorsed by the Cochrane Collaboration was assessed by two reviewers focusing on six bias domains: sequence generation, allocation concealment, blinding, incomplete outcome data, selective outcome reporting, and other possible bias. We constructed a risk of bias table and showed the risk of bias of included studies with a “low” (green), “unclear” (yellow), or “high” (red) risk of bias. Disagreements were also resolved through discussion with a third reviewer. The results of the quality assessment were reported using Review Manager 5.3 (software).

### Selection of studies and data extraction

Two researchers completed literature screening independently in strict accordance with the inclusion and exclusion criteria. Endnote X9 software was used to delete duplicate articles. Literature screening and information extraction were carried out by reading the title, abstract, and full text. In case of disagreement, the two researchers negotiated and discussed, with a third reviewer if necessary. The extracted information mainly included researcher name, publication time, country, number of intervention group/control group, age of subjects, gender ratio, intervention measures, and outcome indicators.

### Risk of bias of included studies

The Cochrane Manual 5.3.0 was used to evaluate the risk bias of randomized controlled studies, including randomized allocation method, allocation hiding, blindness, data integrity, selective reporting of study results, and other sources of bias.^30^ Each project was evaluated as “high risk of bias” “low risk of bias” and “unclear."

### Data analysis

Meta-analysis was performed using RevMan 5.3. Standardized mean difference (SMD) or mean difference (MD) were used as effect analysis statistics for continuity variables. Risk ratio (RR) and odds ratio (OR) were effect analysis statistics, and each effect size provided 95% CI. Heterogeneity between study results was included in combination with X^2^ test and *I*^2^ analysis. If *P* ​> ​0.1，*I*^*2*^ < 50 indicated good homogeneity, and fixed effect model was adopted. If *P* ​< ​0.1，*I*^*2*^ ≥ 50％, indicating obvious heterogeneity, random effect model was adopted. Clinical and methodological heterogeneity was characterized by meta-regression, subgroup analysis, sensitivity analysis, or qualitative characterization, depending on the situation. Egger's method was used to test the publication bias of the included studies. If *P* ​> ​0.05, indicating no significant publication bias, if *P* ​< ​0.05, and vice versa. Since all of the studies were continuous variables and the assessment tools for each test index were different, standardized mean square was used as the effect index, and 95% CI was the effect analysis statistic.

## Results

### Search results

**Fig. 1:**
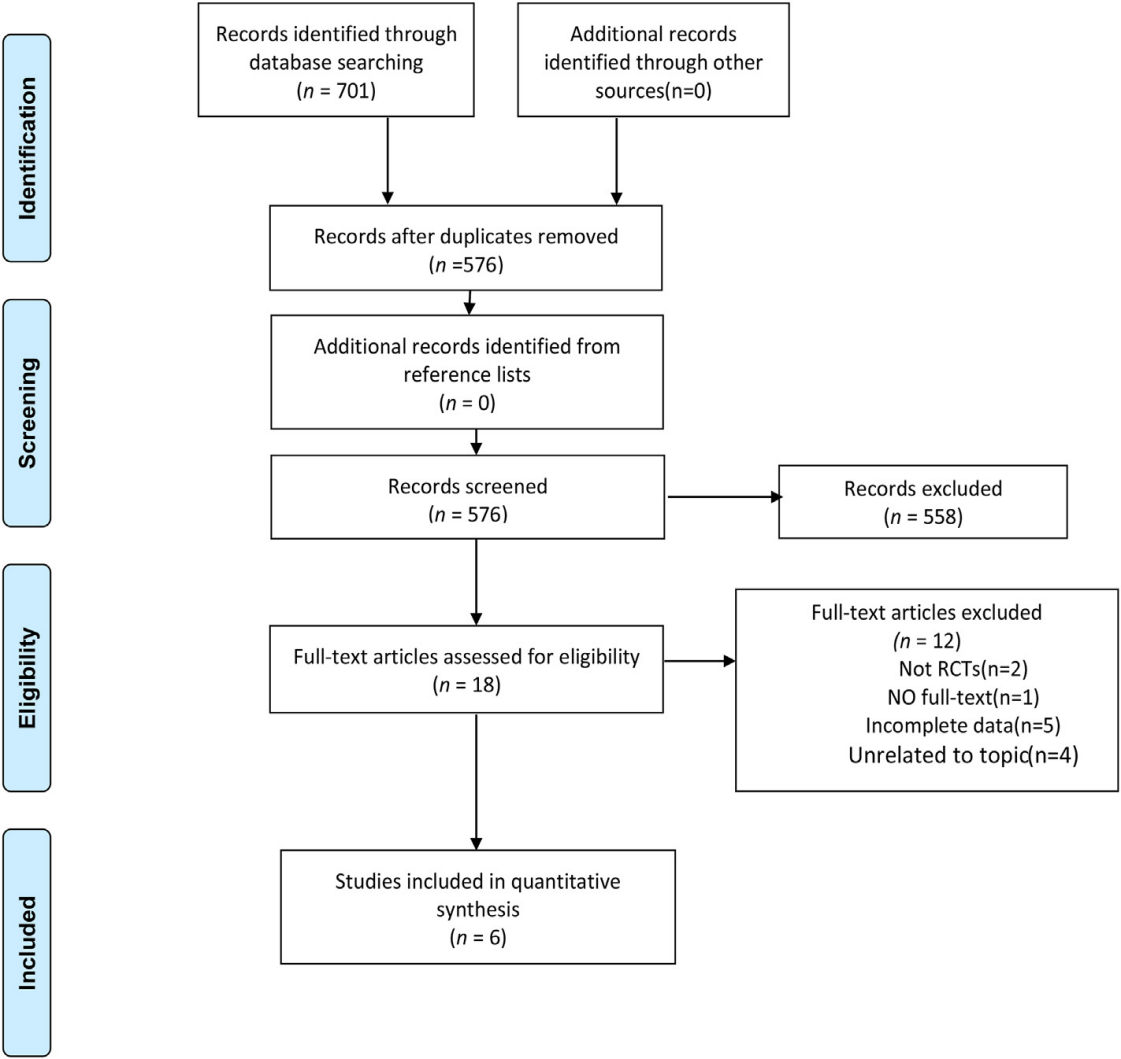
PRISMA search flow diagram.

A total of 697 related articles were retrieved, including 690 articles in English and seven in Chinese. Finally, six articles in English were included after reviewing the full content of the papers. PRISMA flow diagram of study selection process was shown in Fig. 1.

### Study characteristics

The total sample size of the six included studies was 379 cases, including 206 cases in the experimental group and 173 cases in the control group. The basic characteristics of the included studies are shown in Table 1.

**Table 1:** Study characteristics (*n* ​= ​379).

Study/Year	Setting	No. (T/C)	Mean age/years	Gender (M/F)	Time since diagnosis (mo)	Intervention	Intervention situation	Outcomes/measure
Gerçeker et al/2021^5^	Turkey	21/21	T: 6-17 C: 6-17	13/8 13/8	NA	T: VR① C: standard care	Started 2–3 ​min before the procedure and continued until the procedure of port with Huber needle was completed	Pain: Wong-Baker Faces pain scores Anxiety: CAM-S Fear: CFS
Hundert et al/2022^31^	Canada	20/20	T: 12.1 ​± ​3.0 C: 12.6 ​± ​3.6	13/7 12/8	T: 9.9 ​± ​10.3 C: 11.6 ​± ​10.5	T: VR② C: iPad	SCP needle insertions	Pain: NRS Distress: NRS Fear: CFS
Semerci et al/2021^32^	Turkey	35/36	T: 11.69 ​± ​3.36 C: 11.67 ​± ​3.55	16/19 19/17	T: 20.97 ​± ​14.30 C: 20.51 ​± ​20.67	T: VR ③ C: standard care	Venous port access	Pain: Wong–Baker Faces Pain Rating Scale
Sharifpour et al/2021^33^	Iran	15/15	T: 14.8 ​± ​2.4 C: 15 ​± ​1.85	NA	NA	T: VR④ C: standard care	Chemotherapy	Pain: MPQ Anxiety: PASS Nausea: PCS
Tennant et al/2020^34^	Australia	61/29	11.59 ​± ​3.34	50/40	255.66 ​± ​338.67 days	T: VR⑤ C: Ipad	10 ​min when rest in bed	Pain: VAS Anxiety: VAS Nausea: VAS
Wong et al/2021^35^	China	54/54	T: 10.5 ​± ​3.8 C: 10.2 ​± ​3.5	30/24 22/32	T: 12.7 ​± ​8.9 C: 13.9 ​± ​15.8	T: VR⑥ C: standard care	5 ​min before and during peripheral Intravenous cannulation	Pain: VAS Anxiety: CSAS-C

T, treatment; C, control; M, male; F, female; NA, not available; VR, virtual reality; No., number; mo, month.

CAM-S, The Children's Anxiety Meter-State; CFS, Child Fear Scale; NRS, 11-point Numeric Rating Scale; MPQ, The McGill Pain Questionnaire; PASS, The pain anxiety symptoms scale; PCS, The pain Catastrophizing Scale; VAS, visual analogue scale; CSAS-C, the short form Chinese version of the state Anxiety Scale for Children.

T: VR① Watch Rilix VR/Ocean Rift/watch in the eyes of animal.

T: VR② The VR intervention used auditory and visual stimuli (a game which consisted of aiming rainbow balls at sea creatures as they explored an underwater environment in search of treasure) to distract the participant before, during, and after the SCP needle insertion. C: Watch a video on an iPad.

T: VR③ The rollercoaster video in which a roller coaster speeds up and slows down in the forest accompanied by slow music.

T: VR④ Ocean journey.

T: VR⑤ Participants viewed one of three 10-min virtual simulation experiences, including simulated travel to Australian national parks, Australian zoos, or global city tourist spots C: Ipad and over ear headphones to deliver identical content.

T: VR⑥ VR cartoons.

### Quality assessment

**Fig. 2:**
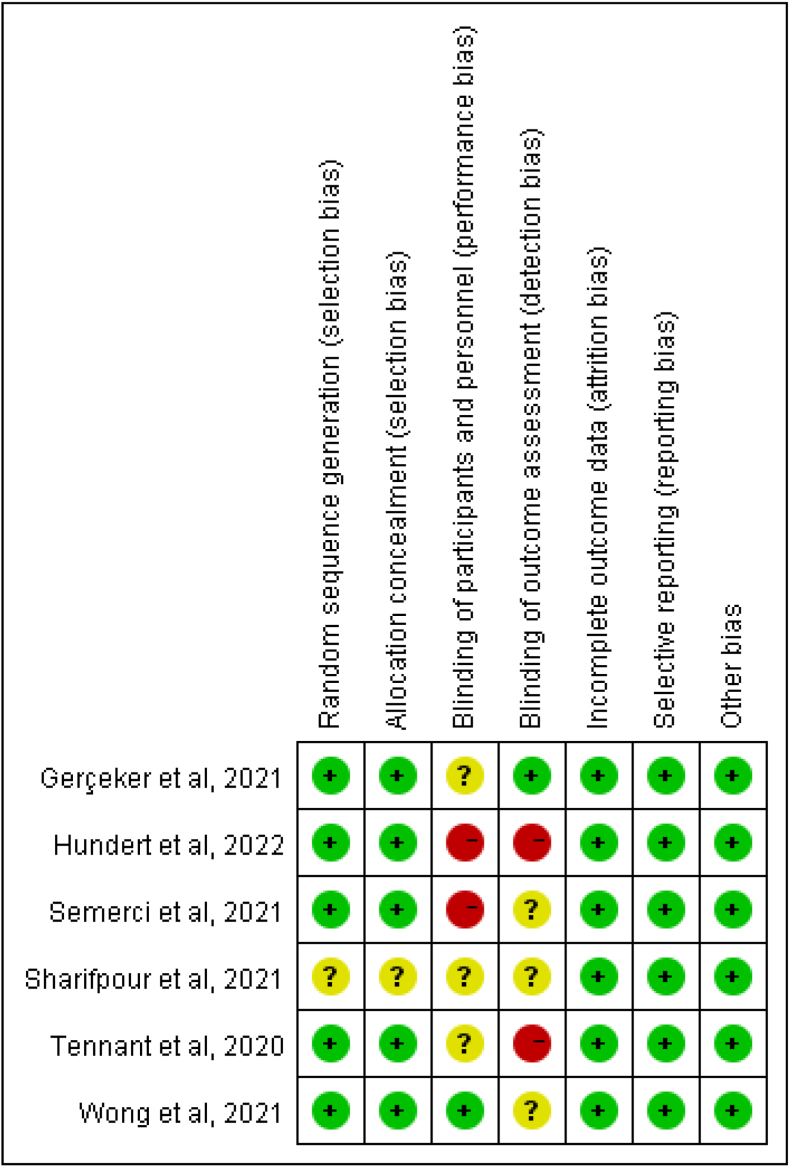
Risk of bias summary.

The methodological quality of six studies was shown in Fig. 2. A Five studies^5^^,^^31^^,^^32^^,^^34^^,^^35^ reported the random method and allocation method, and one study^33^ mentioned the random allocation but did not specify the method. Since the intervention target was children, only one^35^ was successfully implemented to blind the researcher and the subject, which may result in biased reporting of the researcher and result bias. Although the intervention methods of the six included articles were different, all the intervention measures included in this study were VR technology.

### Efficacy analysis

#### Pain

Pain was measured in six trials involving 379 patients, using VAS, Wong-Baker Faces pain rating scales, NRS, and MPQ to assess. The measured overall effects demonstrated the positive role of VR on pain for oncology pediatrics (MD ​= ​−4.82, 95% CI [−7.74, −1.89], *P*＜0.01; *I*^*2*^ ​= ​95%, *P*＜0.01).

**Fig. 3:**
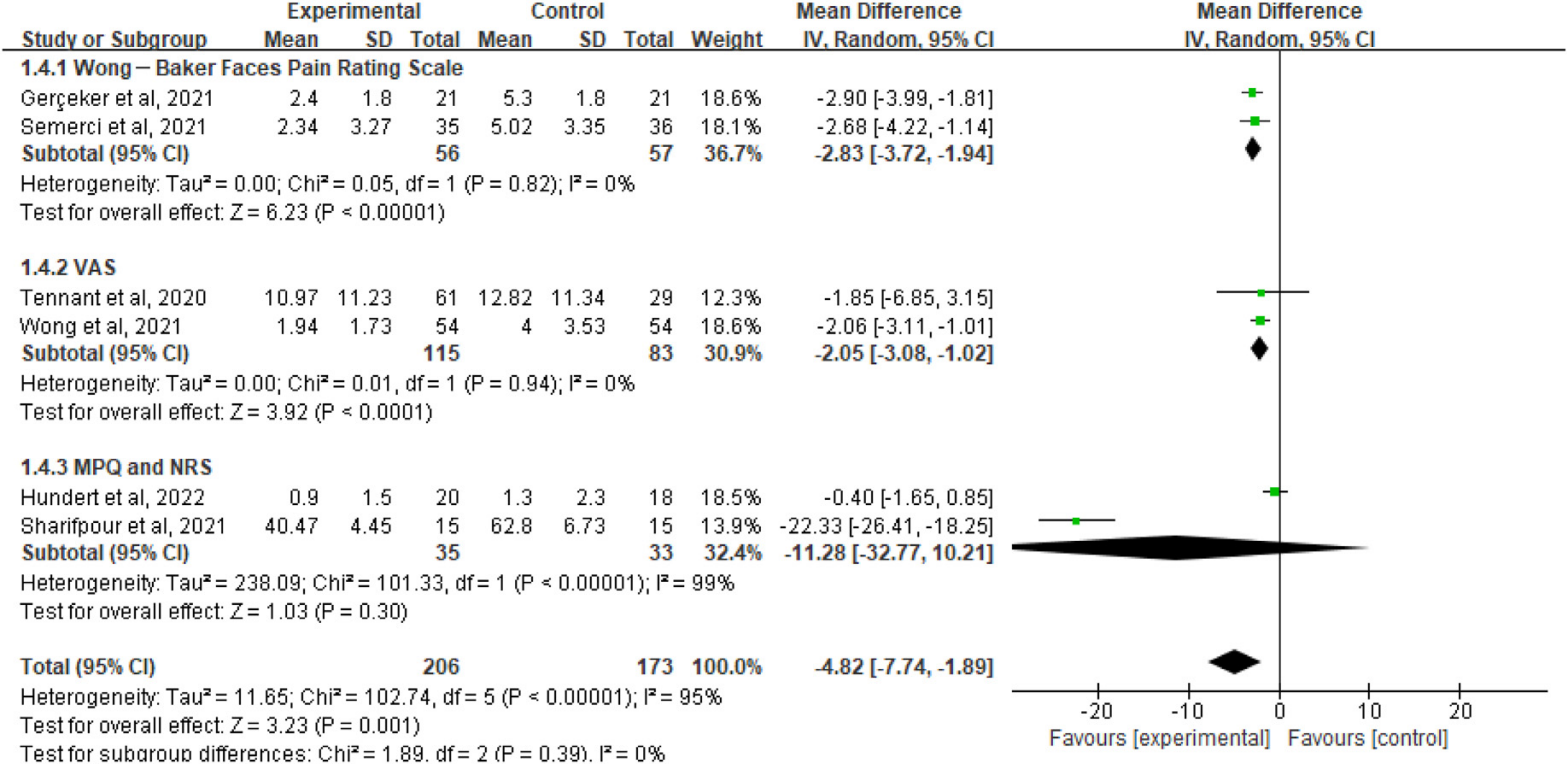
Forest plot for virtual reality on pain.

A subgroup analysis was conducted to determine factors affecting heterogeneity using the different scales, and Sharifpour^33^ and Hundert^31^ were the main sources of heterogeneity. Two studies^5^^,^^32^ assessed the effects using Wong–Baker Faces pain rating scores on pain relief which showed favorable effect (MD ​= ​−2.83, 95% CI [−3.72,1.94], *P*＜0.01) with low heterogeneity (*I*^*2*^ ​= ​0%, *P*＜0.01). Two studies^34^^,^^35^ assessed pain using VAS, for which a consistent effect size was found (MD ​= ​−2.05, 95% CI [−3.08, -1.02], *P*＜0.01) with low heterogeneity (*I*^*2*^ ​= ​0%, *P*＜0.01). Two studies^31^^,^^33^ determined the effects of VR on pain using MPQ and NRS (MD ​= ​−11.28, 95% CI [−32.77, 10.21], *P* ​= ​0.3) with substantive heterogeneity (*I*^*2*^ ​= ​99%, *P*＜0.01 (Fig. 3).

**3.4.2 Anxiety:** A total of 310 participants in five trials^5^^,^^31^^,^^33^, ^34^, ^35^ were included to assess to the effects of VR on children with cancer by using CSAS-C, PASS, NRS, The State Anxiety Scale for Children, and SCAS. The results showed significant effects between VR group and the control group (SMD ​= ​−1.47, 95% CI ​[−2.46, −0.48], *P*＜0.01; *I*^*2*^ ​= ​92%, *P*＜0.01) with the random model. By excluded two studies by Sharifpour^33^ and Tennant,^34^ a significant reduction in heterogeneity (*I*^*2*^ ​= ​0%) was observed.

**Fig. 4:**
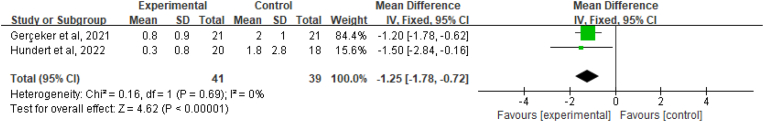
Forest plot for virtual reality on fear.

**3.4.3 Fear:** The effects of VR on fear using The Child Fear Scale (CFS) were reported in two trials^5^^,^^31^ with 80 patients. Based on a fixed-effects model, the analysis revealed significant effects of VR for fear (MD ​= ​−1.25, 95% CI [−1.78, 0.72], *P*＜0.01) with no substantial heterogeneity (*I*^*2*^ ​= ​0%, *P* ​= ​0.69), as shown in Fig. 4.

#### Publication bias

The number of included studies for each outcome indicators in this study was less than 10, so it was not possible to draw a funnel plot to describe and analyze publication bias, but the possibility of publication bias in some included studies could not be ruled out.

## Discussion

To the best of our knowledge, this is the first systematic review and meta-analysis to ascertain the effects of VR for reducing pain, anxiety, and fear of pediatric cancer patients. Based on six studies for pain, five studies^5^^,^^31^^,^^33^, ^34^, ^35^ for anxiety, two studies^5^^,^^31^ for fear, this meta-analysis showed VR to be an effective tool to diminish pain (MD ​= −4.82), anxiety (SMD ​= ​−1.47), and fear (MD ​= ​−1.25) of children with cancer during a range of medical procedures or pain crisis. Our studies supported the recommendations for the use of VR in Loeffen et al.‘s clinical practice (2020).^4^ Linked to the findings of previous reviews, we acknowledge the positive role of VR in procedural pain, anxiety, and fear management.

Six studies^27^, ^28^, ^29^, ^30^, ^31^, ^32^ were included to summarize the evidence on the effectiveness of VR intervention in reducing pain during painful medical procedure or pain crisis in pediatric oncology patients. This meta-analysis suggests that VR may be considered to be a viable intervention in a pediatric oncology population for reducing pain. However, the included articles involved various types of VR and application scenarios, it is not possible to determine which scenes VR could be the most effective tool to diminish pain. A meta-analysis by Oliver Czech suggests that VR has the potential to become an important tool in young adults undergoing needle related procedures in a variety of medical settings.^6^ It suggests the usefulness of VR during painful procedures in children.

Pain is a common experience for children with cancer, and pain relief is always a major part of treatment.^36^ There are many ways to relieve pain, both pharmacological and nonpharmacological. VR is a nonpharmaceutical technique for pain relief, which immerses children in a vivid, interesting, and realistic virtual world through multisensory stimulation such as vision, hearing, and touch. VR fully arouses children's participation enthusiasm, creates effective interaction with the virtual world, diverts attention from adverse stimuli, and ignores aversive stimuli.^23^^,^^37^^,^^38^ When children focused on events other than painful medical manipulation, there was less activation in brain regions associated with pain, such as the thalamus, insula, and the anterior cingulate cortex, local cerebral blood flow associated with processing events is reduced, pain signals to their brains are correspondingly reduced, and children temporarily forget the pain they are experiencing, resulting in a correspondingly lower pain score.^39^

Untreated pain could lead to negative psychology in children. More seriously, it can have a damaging effect on their future pain perception.^40^ Therefore, the use of VR technology intervention is of great significance to the management of pain in pediatric oncology patients. However, it is worth noting that the safety of VR technology has yet to be further verified. More research is also needed on children with cancer cope with procedural pain or during a pain crisis.

Different scales were used for subgroup analysis according to the study, and Sharifpour^33^ and Hundert^31^ were the main sources of heterogeneity. This suggests that we could use the same evaluation tools to assess as much as possible in the future, for it may contribute to a more specific conclusions that VR is an effective intervention in reducing pain for pediatric oncology patients.

One possible reason of significant reduction in the symptoms of anxiety is that coupled with weak physical resistance and susceptibility to infection, most children with cancer have relatively few opportunities to participate in leisure activities.^41^ Restricted in hospital, children were lack of opportunities for children to play and their anxiety of hospitalization increased.^42^ It is worthwhile to note that the studies used different modalities of VR to relieve anxiety. Likewise, studies varied as regard the cancer types and control group, which might thus lead to substantial heterogeneity. For our studies utilized VR video or VR games as intervention, iPad video or standard care as control group. We have also noted the fact that different outcome measures were used to evaluated the anxiety levels in our review, including CAM-S, NRS, PASS, VAS, and CSAS-C. Our study did not constitute irrefutable evidence that VR is effective in reducing medical anxiety in children with cancer experiencing pain for the heterogeneity above. Heterogeneity is mainly derived from Sharifpour^33^ and tennant,^34^ and this heterogeneity cannot be explained by subgroup analysis. Because Sharifpour^33^ and Tennant's^34^ intervention scenarios were one of chemotherapy and one was lying bed, and they didn't specify what kind of pain treatment they were undergoing. Sharifpour^33^ did not specify how long the children had been diagnosed with cancer, which could be a source of heterogeneity. Future studies should contain more consistent condition involves VR types, medical setting, and outcome measures. In addition, more details about stage of cancer are needed.

The results of this study showed that VR technology can relieve fear in children with cancer compared to standard care group, which may hint the potential benefits of immersive VR. This may be due to the significant fear of painful medical procedures in children with cancer who already face the disease burden of a series of treatments.^5^^,^^16^ Coupled with the sense of presence provided by 3D glasses, VR videos included the multidimensional visual, and sound experience. It creates an ever-changing experience that requires a high degree of attention to process incoming stimuli.^43^ In addition, the VR head-mounted display covers the medical environment where children seek medical treatment, reducing children's fear of the environment, shielding the environment from bad sound stimulation, and creating a warm and happy medical atmosphere for children.^44^

### Limitations

There are some limitations in this study. First of all, the outcome measurement tools, types of cancer, types of VR, and intervention scenarios of children included in the studies were various, which may increase the heterogeneity of the combined results. Second, some randomized controlled trials were included without double blindness or allocation hiding, which had a certain impact on the quality of the articles. Finally, funnel plot was not drawn, which may have potential publication bias.

## Conclusions

Despite the limitations mentioned above, this systematic review and meta-analysis found that VR can lessen pain, anxiety, and fear. VR could be encouraged in the clinical care of children with cancer. Future well-designed randomized trials with identical scales and specific comparisons of different types of cancer should help draw definite conclusions of VR on pain, anxiety, and fear in pediatric oncology patients.

## Author contributions

Zhi Cheng, Xin Liu, Yiju Shen, Hong Weng: Conceputalization, Methodology, Software. Zhi Cheng, Shanzhen Yu: Writing original draft preparation. Shanzhen Yu, Wen Zhang: Supervision. Zhi Cheng, Shanzhen Yu, Wen Zhang: Writing – reviewing and editing.

## Declaration of competing interest

None declared.
