# Supplementary material for: Virtual reality for pain and anxiety of pediatric oncology patients: A systematic review and meta-analysis
Source: Asia Pac J Oncol Nurs. 2022 Sep 28;9(12):100152. doi: 10.1016/j.apjon.2022.100152 (PMC9640949; doi:10.1016/j.apjon.2022.100152)
Supplement: Multimedia component 1 [file mmc1.pdf]

INPLASY PROTOCOL

To cite: Cheng et al. Virtual reality for pain and anxiety of pediatric oncology patients: A systematic review and meta-analysis. Inplasy protocol 202230108. doi: 10.37766/inplasy2022.3.0108

Received: 21 March 2022

Published: 22 March 2022

Corresponding author:
Shanzhen Yu

shanzhen_1209@163.com

Author Affiliation:
Zhongnan Hospital of Wuhan University.

Support: **None.**

Review Stage at time of this submission: **Formal screening of search results against eligibility criteria.**

Conflicts of interest:
None declared.

Virtual reality for pain and anxiety of pediatric oncology patients: A systematic review and meta-analysis

Cheng, Z[1]; Yu, S[2]; Zhang, W[3]; Liu, X[4]; Shen, Y[5]; Weng, H[6].

Review question / Objective: **Effects of virtual reality technology on pain, anxiety, fear and vomiting in children with cancer**
Condition being studied: **Cancer is a common and debilitating disease and a leading cause of death among children in both developed and developing countries. The estimated global incidence of childhood cancer in 2015 was 396,670 cases. In recent years, due to the improvement of diagnostic methods, the rate of early diagnosis of childhood tumors has been significantly increased. At present, the 5-year survival rate of children under 15 years old in the United States has reached 70% or higher, and only a few children have a 5-year survival rate of 60%-70%. The overall treatment has made significant progress.However, due to the severity of the disease, the unpredictability of the prognosis of the disease, various treatments have brought adverse life experience to the children.At present, virtual reality technology has been widely used in perioperative anesthesia induction, burns, stomatology, autism, brain injury rehabilitation and other fields. Studies have shown that virtual reality technology can improve pain and anxiety in children with cancer. However, some studies show that there is no significant difference in the effect of virtual reality technology intervention compared with other technology interventions. Therefore, this study used meta-analysis to evaluate the effect of virtual reality technology on relieving pain and anxiety related to children with cancer, in order to provide scientific evidence-based basis for relieving pain and anxiety in clinical nursing practice.**

INPLASY registration number: This protocol was registered with the International Platform of Registered Systematic Review and Meta-Analysis Protocols (INPLASY) on 22 March 2022 and was last updated on 22 March 2022 (registration number INPLASY202230108).

INTRODUCTION

Review question / Objective: **Effects of virtual reality technology on pain, anxiety, fear and vomiting in children with cancer.**

Condition being studied: **Cancer is a common and debilitating disease and a leading cause of death among children in both developed and developing countries. The estimated global incidence of**

Cheng et al. Inplasy protocol 202230108. doi:10.37766/inplasy2022.3.0108 Downloaded from https://inplasy.com/inplasy-2022-3-0108/

childhood cancer in 2015 was 396,670 cases. In recent years, due to the improvement of diagnostic methods, the rate of early diagnosis of childhood tumors has been significantly increased. At present, the 5-year survival rate of children under 15 years old in the United States has reached 70% or higher, and only a few children have a 5-year survival rate of 60%-70%. The overall treatment has made significant progress.However, due to the severity of the disease, the unpredictability of the prognosis of the disease, various treatments have brought adverse life experience to the children.At present, virtual reality technology has been widely used in perioperative anesthesia induction, burns, stomatology, autism, brain injury rehabilitation and other fields. Studies have shown that virtual reality technology can improve pain and anxiety in children with cancer. However, some studies show that there is no significant difference in the effect of virtual reality technology intervention compared with other technology interventions. Therefore, this study used meta-analysis to evaluate the effect of virtual reality technology on relieving pain and anxiety related to children with cancer, in order to provide scientific evidence-based basis for relieving pain and anxiety in clinical nursing practice.

METHODS

Participant or population: Participants aged 18 years or younger clinically diagnosed as any types of cancer identified by pathological or cytological diagnosis.

Intervention: Intervention groups received virtual reality in regardless of combining other treatments or not, without restricting on VR's duration, frequency and modalities. The control group undergo standard care, no intervention or some treatment differed from VR.

Comparator: Comparing virtual reality with standard care. Comparing virtual reality with ipad intervention.

Study designs to be included: Randomized controlled trials.

Eligibility criteria: 1.Types of studies. Randomized controlled trials were included with whom publication language is restricted to English or Chinese.2.Types of participants. Participants aged 18 years or younger clinically diagnosed as any types of cancer identified by pathological or cytological diagnosis. 3.Types of intervention. Intervention groups received virtual reality in regardless of combining other treatments or not, without restricting on VR's duration, frequency and modalities. The control group undergo standard care, no intervention or some treatment differed from VR.4.Types of outcome measures. The primary outcome was pain and anxiety. The secondary outcome included fear and nausea.

Information sources: Two reviewers independently searched the following databases: Pubmed, Embase, Cochrane library, Web of science, the Chinese Biomedical Database (CBM), the China National Knowledge Infrastructure (CNKI), VIP Journal Integration Platform and Wanfang Med Online, without language restriction, from inception to February 22,2022.

Main outcome(s): The primary outcome was pain and anxiety.

Additional outcome(s): The secondary outcome included fear and nausea.

Quality assessment / Risk of bias analysis: The risk of bias of the included studies was assessed independently by two researchers (zhi and Xingxing) using the Cochrane Collaboration System Evaluation Manual (version 5.1.0) criteria. The risk of bias scale included the following five entries: Random sequence generation (selection bias), Blinding of participants and personnel (performance bias), Blinding of outcome assessment (detection bias), Incomplete outcome data (attrition bias) and Selective reporting (reporting bias). Based on the information extracted from each eligible trial, each potential source of

Cheng et al. Inplasy protocol 202230108. doi:10.37766/inplasy2022.3.0108 Downloaded from https://inplasy.com/inplasy-2022-3-0108/

bias will be classified as high risk, unclear or low risk, and any disagreement will be determined by the third author (Shan zhen).

Strategy of data synthesis: Meta-analysis was performed using RevMan 5.3. Standardized mean difference (SMD) or mean difference (MD) were used as effect analysis statistics for continuity variables. Risk ratio, Relative Risk and Odds ratio (OR) were effect analysis statistics, and each effect size provided 95% CI. Heterogeneity between study results was included in combination with X^2 test and I^2 analysis. If P>0.1, I^2<50 indicated good homogeneity and fixed effect model was adopted. If P<0.1, $I^2 \geq 50\%$, indicating obvious heterogeneity, random effect model was adopted. Clinical and methodological heterogeneity was characterized by meta-regression, subgroup analysis, sensitivity analysis, or qualitative characterization, depending on the situation. Egger's method was used to test the publication bias of the included studies. If P>0.05, indicating no significant publication bias, if P<0.05, and vice versa. Since all of the studies were continuous variables and the assessment tools for each test index were different, standardized mean square was used as the effect index and 95%CI was the effect analysis statistic.

Subgroup analysis: A subgroup analysis was conducted to determine factors affecting heterogeneity using the different scales.

Sensitivity analysis: Sensitivity analysis was carried out using an exclusion-by exclusion method.

Language: Without restriction of language.

Country(ies) involved: Hubei province, China.

Keywords: Virtual reality, Pediatric oncology patients, Pain and anxiety, Meta analysis.

Contributions of each author:
Author 1 - zhi cheng.
Email: chengzhi503@yeah.net
Author 2 - Shanzhen Yu.
Email: shanzhen_1209@163.com
Author 3 - Wen Zhang.
Email: wzhang115@163.com
Author 4 - Xinxin Liu.
Email: 270962057@qq.com
Author 5 - Yijin shen.
Email: 1747637867@qq.com
Author 6 - Hong Weng.
Email: wengh92@163.com

Cheng et al. Inplasy protocol 202230108. doi:10.37766/inplasy2022.3.0108 Downloaded from https://inplasy.com/inplasy-2022-3-0108/
